# The recovery experience of people who were sex trafficked: the thwarted journey towards goal pursuit

**DOI:** 10.1186/s12914-019-0185-7

**Published:** 2019-01-22

**Authors:** Roderik F. Viergever, Nicki Thorogood, Tamara van Driel, Judith RLM Wolf, Mary Alison Durand

**Affiliations:** 1 Research4health, Utrecht, the Netherlands; 2 CoMensha, Amersfoort, the Netherlands; 3 Department of Health Services Research and Policy, London School of Hygiene and Tropical Medicine, Tavistock Place, London, WC1H 9SH UK; 4 Department of Public Health, Environments and Society, London School of Hygiene and Tropical Medicine, Tavistock Place, London, WC1H 9SH UK; 5 Impuls, the Netherlands Center for Social Care Research, Radboud university medical center, Nijmegen, the Netherlands

**Keywords:** Human trafficking, Sex trafficking, Vulnerable groups, Sex work, Prostitution, Slavery, Qualitative research, Migrants, Exploitation, Shelter, Violence, Social care, Health

## Abstract

**Background:**

In 2010, a shelter programme was established in the Netherlands to provide social and health services for trafficked people. This article describes how service users in this programme conceptualized and experienced their own process of recovery.

**Methods:**

In 2012, 14 people of non-Dutch nationality who had been trafficked for the purpose of sexual exploitation were interviewed at all three shelters of the programme. Data analysis followed a grounded theory approach.

**Results:**

Participants felt a strong need to turn over a new leaf in life, leaving negative experiences of the past behind and moving towards a life with a job, a family and friends. In contrast with their willingness to work towards realizing that future, they experienced a lack of autonomy and a thwarted sense of agency in redressing their present situation. Together with the ostracized nature of their place in Dutch society this left them ‘in limbo’: a feeling of standing still, while wanting to move forward. This led participants to find it more difficult to deal with problems related to their pasts and futures. They particularly appreciated Dutch language training, vocational skills training and opportunities for volunteer work.

**Conclusions:**

Participants exhibited a strong desire to fulfil the basic psychological needs of competence, relatedness and autonomy, but were thwarted in pursuing these goals. Seemingly against all odds, while faced with several external regulators that limited their agency to change their situation, participants found ways to pursue these goals, through their enthusiasm for activities that helped them get closer to their envisioned futures (language and skills training and volunteer work). Identifying pathways toward attaining their goals allowed them to hope for a better future. That hope and pursuing their goals helped them to cope with the problems of their past and their worries about the future. Therefore, to facilitate service users’ recovery in a post-trafficking setting, there is a need to provide them with opportunities to hope for, pursue and attain their personal goals within the structural boundaries of their situation. A future-orientated, strengths-based approach towards service provision and responsive and supportive environments help to do this.

**Electronic supplementary material:**

The online version of this article (10.1186/s12914-019-0185-7) contains supplementary material, which is available to authorized users.

A layperson summary and policy brief for this article are available in Additional file 1.

## Background

The violence and abuse that accompany human trafficking may result in health problems and social problems such as stigma and shame for people who have been trafficked [1–4]. In the Netherlands, an estimated 6250 people are trafficked each year [5], of whom around a 1000 are identified and around 200 seek social and health services from a shelter [6] (see below *Additional background on making estimates about numbers of trafficked persons*). In 2010, a new national shelter programme for trafficked people was established to provide specialized shelter services for this population: the Categorical Care for People who were Trafficked (Dutch acronym: COSM) programme.

While social and health services for trafficked persons have been established in various countries in North America and Europe, the evidence that exists to base best practices on is extremely limited [3, 7, 8]. Although a range of studies have shed light on the post-trafficking service needs and experiences of trafficked people, most have been published as grey literature and although some studies provide great insight and depth, many are quite limited with regards to services analysis [3, 7, 8].

To increase our knowledge specifically of what service users *themselves* feel the goals of post-trafficking social and health service provision should be, and what approaches to that service provision should be taken to help them achieve those goals, this article explores how service users in three Dutch COSM shelters conceptualize and experience their own process of recovery.

### Additional background on making estimates about numbers of trafficked persons

Each year, 1.2 to 3 million people are trafficked and globally there are 21 to 40 million adults and children who live in conditions that are often referred to as ‘modern-day slavery’ [9–13]. In the Netherlands, an estimated 6250 people are trafficked each year [5]. Making estimates such as these – about the number of trafficked persons in a country or even globally – is highly challenging. The hidden nature of the population makes identification of people who have been trafficked very difficult. Internationally, it is estimated that only 5–10% of these people are identified [9, 10]. That number is much smaller if debt bondage and forms of slavery are included. In the Netherlands a method of making these estimates was newly applied recently, which used data from various registry sources (e.g. police, health providers, social workers, youth care) and combines them using a ‘capture-recapture analysis’, also called ‘multiple systems estimation’. [5] Further improvements to this methodology are desirable: ideally, they would also include estimates about the number of trafficked persons that will never be registered anywhere (these are currently not included in the estimates). This would require incorporating results from surveys about the prevalence of human trafficking among at-risk populations (e.g. migrant labourers or sex workers). Such surveys are regularly conducted but have not been conducted in the Netherlands yet [12]. It would be useful to combine these two methods of estimation; research to do so deserves recommendation for the future.

## Methods

### Informed consent and ethical approval

Acquiring informed consent from victims of human trafficking in a research study is never straightforward. In this research considerable time was devoted to ensuring that informed consent was acquired in an appropriate manner.

In preparation of the research, all the countries where service users of the COSM programme originated from in 2010 were mapped out. Service users came from almost 40 different countries. Their country of birth was most often in Africa (70%), followed by Europe (14%), Asia (8%), and South America (6%) [personal communication T. Van Driel 10 June 2011]. The official spoken languages of these countries were mapped in concordance: 46% of service users came from countries where English is the official language. All other official spoken languages each corresponded to less than 10% of service users. Participants from English language countries did not necessarily speak English (often people speak only local languages). In addition, the composition of the group of service users varied every year [14–16].

The possibilities for acquiring consent from such a *variable* (changing over time) and *varied* (differing per person) population in terms of language were explored. Although consent forms should preferably be written in the preferred language of the participant, it has been suggested that for research with people from multiple language groups where not all languages can be predetermined prior to recruitment, oral translation of consent forms is acceptable [17]. This method for obtaining consent has been used for example in studies with immigrants [18] and is supported by articles that argue that reading out the details of the consent form to participants is acceptable for cross-cultural research [19].

This approach was taken in this study. Information sheets and consent forms were prepared in Dutch and English. If the participant could read Dutch or English, the information sheet and consent form were given to him/her to read. If the participant could not read Dutch or English, the interviewer read out the forms, or the forms were translated with the help of an interpreter into the participant’s preferred language. During this procedure regular checks were made to ensure things were clear and further explanation was provided if things were not clear.

Participants were then asked to sign the consent form. Previous research among victims of human trafficking has shown that, for various reasons, victims are not always willing to sign or mark consent forms even though they do consent to participating in the research [20]. When this occurred, verbal consent was acquired, which was tape-recorded.

These procedures (including the use of verbal consent when appropriate) and the study as a whole were approved by the London School of Hygiene and Tropical Medicine Ethics Committee and by the Medisch Ethische Toetsingscommissie Slotervaartziekenhuis en Reade in the Netherlands.

### Context: the COSM programme

The COSM programme consists of three shelters, two for women and one for men, and at the time of this study had a total of 50 beds. Service users were exclusively adults; > 95% were of foreign nationality. The COSM shelters are a type of crisis shelter and provide shelter for a maximum of 3 months. More information is provided in Additional file 2.

### Research methodology

The research applied a grounded theory approach combined with some aspects of narrative research. The methods of data collection and analysis used are described below.

### Data collection

Interviews were conducted by the first and third author with 14 service users of the COSM programme. Participants were interviewed from 28 February 2012 to 18 October 2012. Participants were generally interviewed twice (two were interviewed three times; one was interviewed once), to allow for the interviewer and the participant to build enough rapport for an interview about sensitive topics. In total, 29 interviews were conducted with 14 participants. Eight participants were interviewed in English, one in Dutch, and five using an interpreter (one in person, the remainder via phone). Participants could choose in which language they wanted the interview to be held and if they wanted to make use of an interpreter or not. Interviews were taped if participants provided consent (50% did so). When taped, the interviews were transcribed by the first author. When not taped, detailed notes were taken by the first author during the interview and were reviewed, synthesized and digitized on the same day. All interviews were conducted on the premises of the shelter where the participant resided at the time of the interview.

To develop the topic guide, the main researcher (RV) discussed with the other researchers which questions would be most likely to solicit results that would aid the achievement of the aim of the study. In these discussions, the results of a literature review were used (chapter 2 in [3]) and the results of pilot interviews conducted with volunteer fellow researchers. The topic guide is provided in Additional file 3.

### Study population

#### Inclusion criteria

The study population consisted of foreign, adults who had been trafficked and who resided in one of the three COSM shelters.

#### Exclusion criteria

Interviews were limited to service users who had resided in one of the COSM shelters for at least 6 weeks. This was done as a safety measure, to make sure people who had very recently exited a trafficking situation and still needed to find their bearings in the shelter were not interviewed (to prevent possible trauma by interviewing people in that early stage).

Also, people who have been trafficked for other purposes than sexual exploitation have commonly had different trafficking experiences and, as a result, have different social and health care needs and experiences, than people who were trafficked for sexual exploitation. Therefore, we opted to limit the interviews to people who had been trafficked for the purpose of sexual exploitation.

#### Population characteristics

Nine female and five male service users were interviewed. Their median age was 26 [IQR 23–31]. Nine study participants were from Africa, three were from Eastern Europe, one was from Asia and one from the Middle East. Seven participants had no children, five had children, and three were pregnant (total is not 14 because one had children and was pregnant). The median time that the study participants had spent in the shelter prior to the first interview was 2.4 months [IQR 2.0–3.5]. As a result, the findings of this study apply particularly to the later stages of service users’ stays in shelters. Please see Table 5.1 in Viergever, 2018, for more information about the participants [3].

#### Sampling method

Potential participants were most often suggested for inclusion in the study by employees of the shelter; sometimes the primary author suggested participants based on observations in the shelter. The employees were asked to list adult, foreign service users trafficked for the purpose of sexual exploitation. They often suggested service users who would be able to voice their views about the services provided. Which service users would participate in the study was decided upon in discussion between the primary author and the employees of the shelter. This is necessary for this type of research, for several reasons, the main two being safety (to make sure we did not interview service users who were not ready for an interview) and acceptability (to make sure the research was acceptable to the shelter and its employees). The first author always checked potential participants against the inclusion and exclusion criteria of the study and against a list of previous participants and their regions of origin: the purpose of this was to generate a sample that was diverse in terms of regions of origin (i.e. potential participants who were from regions (e.g. Africa, Middle-East, Asia, Europe) from which no one was interviewed yet were given preference over others).

### Data analysis

Data analysis took a grounded theory approach, following the stage of familiarization, open coding, axial coding and selective coding [21, 22]. In addition, some elements of narrative analysis [23, 24] were incorporated, by writing narratives about participants [25]. Writing these narratives was an additional way of becoming familiar with the data [25].

A more detailed description of the methods for data analysis is provided in Additional file 4.

### About the study team and its relation to the shelters

At the time of data collection and analysis all researchers involved in this study were independent from the shelters and other social and health care providers who provided care. The principal investigator, RFV, now works for CoMensha, the Dutch national coordinating centre for human trafficking. This is not a shelter, but an organization that, among others, coordinates care provision (e.g. making sure trafficked persons find appropriate shelters, social care, health care and legal care).

### Terms

This article uses several terms, such as ‘health’, ‘recovery’, ‘victim’ and ‘vulnerability’. Here below, our definitions of these terms are described.

#### Health

The concept ‘health’ was defined by the World Health Organization (WHO) in 1946 as “a state of complete physical, mental and social well-being and not merely the absence of disease or infirmity” [26]. Since then, this definition has been a matter of debate [27–29]. Although most agree that “the conjunction of the physical, psychological, and social remains powerfully relevant to this day” some have criticized the static conceptualization of ‘health’ in the WHO definition [30]. They have expressed support for what was first described by Canguilhem in 1943, that there are no normal or abnormal states of health, but that health should be defined as “the ability to adapt to one’s environment” [30]. Huber has recently advanced these ideas and proposed the following definition of health: “The ability to adapt and self manage in the face of social, physical and emotional challenges” [27].

For people who have been trafficked, a range of potential social problems (such as social stigma, isolation, and difficulties with finding work and education) lies central to any potential physical and mental health problems and influences strongly their post-trafficking service experiences [1, 2]. As Huber et al.’s definition of health allows for an inclusive view of the broad spectrum of challenges that trafficked people face, this definition was adopted for the research.

#### Health consequences of human trafficking

The health consequences of human trafficking are discussed and reviewed extensively elsewhere [3]. A publication by Zimmerman that is particularly useful in considering the health consequences of human trafficking deserves explicit mentioning in this section about terms. She writes: “The health risks, consequences, and barriers to services for trafficked women are similar to those experienced by other marginalised groups, including:

1. migrant women;
2. women experiencing sexual abuse, domestic violence, or torture;
3. women sex workers; and
4. exploited women labourers.” [1]

In this article, comparisons to other vulnerable populations with overlapping needs profiles are drawn, especially to these groups.

#### Victims or ‘trafficked persons’

Use of the term ‘victim’ when referring to trafficked persons has been criticized for several reasons. Most importantly: continuously referring to trafficked persons as ‘victims of human trafficking’ puts a label on trafficked people. By continuously calling them victims, we may convince ourselves (or worse – them!) that a victim is all they are and forget what trafficked people are above all – people; people who have experienced something bad and potentially traumatic. Continuously putting the label of victim on this group implies powerlessness and fails to recognize not only the survival and coping strategies that a trafficked person may have developed during their time of exploitation, but also how strong people can be during a time of recovery and thereafter [31]. Arguably most importantly, many trafficked persons do not consider themselves to be victims. Referring to them as such can be confusing and potentially judgmental, and may even result in inappropriate intervention strategies [32]. The term ‘survivor’ has been suggested as a more suitable alternative [33]. However, this term also has its problems. Most importantly, its use across the scientific discourse on human trafficking is not consistent. Some talk about the transition of victim to survivor in the context of post-trafficking recovery, suggesting that one starts as a victim and recovers to be a survivor [34]. Others explain that during the long periods of abuse, violence, and rape that can occur in trafficking situations one ceases to be a victim at some point and becomes a survivor, using the term to describe a change in psychological state that results from a coping strategy [35]. Yet others refer to survivors simply as those who have exited a trafficking situation [36].

Therefore, this publication make use of the terms ‘trafficked persons’ and ‘trafficked people’.

#### Vulnerability

This article makes mention of ‘vulnerable groups’. The definition for vulnerability that the research adheres to is the following definition of vulnerability in the context of health: “Vulnerable populations are those at risk at any particular point in time for unequal opportunity to achieve maximum possible health and quality of life” [37]. The “unequal opportunity” that may lead to vulnerability can be caused by either different or increased health care needs or by decreased access to prevention or care [37–39].

#### Recovery

While the term ‘recovery’ is sometimes used as synonymous with recovering from some specific problem, such as from mental health problems or addiction, the use of the term in this article is broader. The definition of ‘recovery’ that it adheres to is: “The act or process of returning to a normal state after a period of difficulty” [40]. This broader definition is more appropriate for the research because, as the above definition for health, it allows for an inclusive view of the spectrum of challenges that trafficked people face after having exited a trafficking situation.

It has been noted that recovery is a personal journey, different for everyone, that it can ànd does occur without professional intervention, and that it emphasizes the role of the subject as a self-determining agent of change [41, 42]. These descriptions fit well the view of recovery that this research adheres to.

## Results

### The core phenomenon: a thwarted journey towards goal pursuit

In their stories, participants described different temporal states of ‘self-representation’ [43]: they frequently compared who they were now, to who they were in the past and who they envisioned themselves to be in the future. For example, when one participant was asked why she was in the shelter she replied: “I need help and support, also on the psychological level, to re-find myself and to recover. What I also need is guidance in concrete things, steps that are needed to be able to live a safe life in this country.” As this quote exemplifies, participants also attributed value to the different temporal states of the self (‘self-evaluation’): they generally spoke about (parts of) the past negatively, about the future potentially positively, and about the present as being a transitional state, an ‘in-between state’. In this in-between state, study participants wanted to move forward and start building their futures, but weren’t always able or allowed to do so because of their circumstances: they encountered many barriers in trying to pursue their goals. This became the core phenomenon of this study: participants’ thwarted journey towards goal pursuit.

### Operationalizing the core phenomenon

After identifying this core phenomenon, a more selective coding process was started. People’s experiences with service provision were assessed, their ideas about what care was needed, and the underlying problems that they faced. This allowed for analysis of why people felt thwarted in pursuing their goals. What were the goals that they pursued? What was it that drove them to pursue these goals? What was it that held them back? What were the consequences of being thwarted in their goal pursuit?

### Theoretical framework

Since the temporal comparisons that study participants made were so prominent in their narratives and because these comparisons led to the core phenomenon, a theoretical framework by Gergen was adopted that provided more insight into how and why participants’ narratives were structured this way [43]. Gergen posits that people’s narratives can sometimes serve to unite their pasts and presents, and to signify their envisioned future trajectories. His main article on this subject starts with: “This is a story about stories – and most particularly, stories of self” [43]. Gergen proposes that narratives can be typified by various categories when they contain temporal comparisons, e.g.:

- The stability narrative, “that is, a narrative that links events in such a way that the individual remains essentially unchanged with respect to evaluative position”;
- The progressive narrative, a narrative where the individual links together experiences in such a way that the individual progresses over the evaluative dimension of time;
- The regressive narrative, a narrative where the opposite happens, and experiences are linked in such a way that the individual regresses over the evaluative dimension of time.

This temporal form of self-representation and self-evaluation matched well with how study participants spoke of and attributed value to their pasts, presents and envisioned futures in this study. This framework was adopted *after* the largest part of our analysis (the importance of the temporal dimension of representation and evaluation emerged inductively from our data) because it fit well with our data. The framework is used to present the themes that emerged from our analysis in this Results section of this article.

### The past, the present and the envisioned future

This section provides an analysis of how participants viewed their past selves, their present selves and their envisioned future selves, and of how the representations and valuations of their past and future influenced them in the present.

#### The past

Talking about their trafficking experiences can be difficult for people who were trafficked [44]. Therefore, the interviews did not feature any questions about participants’ pasts unless participants indicated clearly that they felt comfortable talking about it. Rather, general questions were asked, such as: “Could you explain to me why you are here in the shelter?” This allowed participants to decide for themselves what they wanted to speak about.

Many study participants did speak about their pasts. Speaking about the time before they were trafficked, many described that their trafficking situation was not the only difficult experience in their past. People spoke, for example, of: the general dire situation in the home country; working as a sex worker from a young age; experiences of growing up in a broken home; being in a forced marriage; being wrongfully imprisoned; various forms of violence, including rape; and the murder of parents and spouses. However, not all stories were all negative, many started positively. Several then described a defining moment when things turned for the worse, ‘a turning point’, distinguishing between one past state of the self in which all was still good and another when all went downhill to the present. One participant, for example, described how she was happily married and had her own business before her husband was murdered and she had to flee her country, which ultimately led to her being trafficked: “I’m trying to change, to be back to the way I was. But it’s difficult! Sometimes I think I am on my way”. Similarly, another participant described a turning point when his parents were murdered: “Since then, since that day, let me not lie to you, up to now, since that day up to the time I’m talking to you, I don’t … I cannot recognize myself well, you know?” As these quotes exemplify, the turning point was not always the trafficking experience itself; for some the turning point happened before they were trafficked.

Some also spoke freely about what happened during the time they were trafficked. However, when they did, they mostly referred to their experiences in general terms, such as one participant who simply stated that she was abused and another who spoke of “the one who hurt her”. Some spoke about the physical consequences of the abuse but not about the abuse itself. Finally, some indicated that it was not something they could speak about: “They ask me to … they force me to … That one I will not be able to explain, that one”.

Almost all participants came to the Netherlands hoping for a better life. Some were deceived: they expected to get a job (not as a sex worker), thought they were coming on a student visa and were expecting to enrol in a university, or came with someone they had fallen in love with. Some came to the Netherlands themselves to find work, ended up working illegally, and thus became vulnerable to exploitation and trafficking within the Netherlands. And some came to work as a sex worker but ended up being financially exploited or having to pay back a huge ‘debt’ (a common method of exploitation by traffickers).

Participants mostly linked being trafficked to a range of negative emotions, including shame, fear, anger, distrust and depressive feelings. Studies that have focused more specifically on the health consequences of trafficking confirm these findings [1, 4]. However, this was not the case for all: one participant was thankful to her trafficker for having helped her escape from her home country and spoke about him in positive terms, even though he later tried to force her to have an abortion, which she did not want to do. As a result, she fled.

Some participants had resided elsewhere in between exiting the trafficking situation and entering the shelter. Three specific situations were described: staying with a friend; having been in prison; and residing in an asylum seekers’ centre. The second was described as follows by the participant who was in prison: “I was in prison here for five weeks. ( …) Without committing any crime! Because they say I don’t have any documents”. Being in the asylum seeker centre was also described to be a negative experience by one male interviewee, who entrusted someone with details about the physical health problems he was suffering from as a consequence of his sexual abuse. This other person then told other people he was gay, which resulted in him being discriminated against [25]. Asylum seekers centres are frequently reported to be intolerant environments with regards to men who have sex with men [45].

#### The present

In many ways, the present self was an ‘in-between state’ for participants. As one said: “I’m still in recovery, I cannot see outside here!” On the one hand, they had left their past behind them and were now in the shelter, away from the influence of their traffickers. One participant explained, for example, that when she arrived in the shelter she was nervous, but that after that she became more relaxed. Another noted how he was very afraid just after he entered the shelter and had sleeping problems and nightmares, but having been put in touch with a church, he was able to calm down. Thus their entry to the shelter started a transition period that brought safety – not being afraid any longer – and the knowledge that help and support was available when needed.

On the other hand, all participants were not quite where they wanted to be yet either, because many struggled to deal with the mental and physical consequences of their trafficking experiences. One said, for example: “And also, you have to think of the past, what happened to me. Am I going to be like this all the time?” The interviewer asked: “Like what? What do you mean …? ” He replied: “Not happy. With all the things that have happened, I have not been happy. All the time fear”. While this study was not at all designed to conduct any formal evaluation of people’s mental or physical health status, many participants described suffering from depressive feelings, fear and anxiety, sleeping problems, nightmares, suicidal thoughts, headaches and stomach aches and several indicated that they would appreciate the opportunity to speak with a psychologist. Common psychosomatic problems, such as sleeping problems, headaches and stomach aches, were regularly mentioned. Studies by other researchers have shown that these are all common health problems among trafficked people [1, 2, 46]. One participant said: “I am supposed to fall asleep then in one or two hours. But sometimes I lie awake for ten hours, until the morning.” The interviewer asked: “How come you have trouble sleeping?” He said: “When I do something, I am distracted from the past. When I lie down, things come in my head, from different sides.” However, the degree to which participants mentioned suffering from such problems varied. Some did not mention any such problems at all.

As some of the quotes above show, forgetting about the negative events in the past was a daily challenge for many and many expressed a desire to ‘be busy’, which offered distraction. There were several ways to ‘be busy’. Many cited the social interaction in the shelter as a positive influence: “We are here as girls amongst each other, we talk together, joke together, that makes you set your worries aside. It provides distraction.” Another form of distraction was participating in activities organized by the shelter. One shelter offered many activities; the other two very few. Participants in the latter two felt that the lack of activities was a factor in them not being able to stop thinking about the past: “Something happens ( …) and you are reminded. You cannot stop thinking about it. And you cannot forget. You don’t think too much when you’re with people. You think about it when you’re alone in a room. You cannot do anything; that is the problem. Maybe if I had something to do, I would have distraction.” Another participant from the same shelter said: “Maybe if we have opportunity to maybe arrange a football or the opportunity to go for swimming, and other places, meet with other people, you know, that would tend to, to make us forget about the past, maybe. Because if you’re involving in any activity, social activity I mean, you will forget about the past.”

Perhaps the clearest expression of the fact that participants felt they were still recovering from their pasts was that several noted: “I cannot recognize myself”. This appeared to be caused by incongruence in how they viewed themselves. Partially, this stemmed from disbelief, shame and a sense of injustice about how they had let themselves end up in a situation like this. One participant said, for example: “Since I find myself in this place, sometimes I see myself stupid, trust me.” “Why?”, the interviewer asked. “Because … if I’m not stupid, the person does fool me. ( …) Sometimes I wonder, or I imagine, if I am, if this is me […], in this house. If I am the one facing this problem.” For others it stemmed from the fact that they had changed because of what had happened, particularly when they were betrayed or deceived by people they knew: “There are some times when you never trust anyone in your life, because if you consider what you go through, you know you will not trust too many people.”

A second reason why participants felt they were not quite where they wanted to be yet (see beginning of this section), resulting in the feeling of being in an ‘in-between state’, was that there was a gap between the reality of their present lives and where they envisioned themselves to be in the future. However, to be able to explain the effect that envisioning the future had on participants in the present, an explanation of how participants envisioned their futures is first needed.

#### The envisioned future

The futures that participants envisioned for themselves differed; yet, there were similarities in how they spoke about the future. One key characteristic was that all envisioned a future that was *better* than the present. There were similarities to what ‘better’ meant: many mentioned the desire to find a job. For some, particularly for men, this included first getting an education. Furthermore, a characteristic of several participants’ aspirations for a job was that they wanted to do work in which they could help others. Finally, some of the women viewed having a job as necessary to be able to support their children, while some of the men viewed acquiring an education and getting a job as a precondition for finding a wife and establishing a family. For example, one participant noted: “Maybe I finish getting my certificate. ( …) Then maybe I will get a job. Then I will start thinking of getting a family.”

This last comment relates to the second frequently mentioned characteristic of a ‘better’ future: finding a partner and establishing a family. Those who had had to leave their children behind in their home country spoke of them specifically with regard to future plans for their family. Making friends was also viewed as important in achieving a successful life in the Netherlands. “You need their support”, one participant remarked. And according to another: “If you know a little Dutch then at least you’ll be able to communicate. To make friends.”

Furthermore, a spoken wish to have one’s own house was part of a broader theme that constituted the desire for autonomy (i.e. to have control over decisions that concerned their own lives). One participant said, for example: “I would like to go to an independent house, but now I need time. After that we will see.” Small steps in attaining autonomy, such as getting one’s own bank account, were celebrated.

For those from outside the European Union (EU), who made up the majority of the sample, being able to stay in the Netherlands and acquiring a residency permit was a precondition for their envisioned futures and hence a priority concern. In the Netherlands, the right to permanent residency for someone who has been trafficked is reserved by law almost exclusively for those people whose traffickers are prosecuted ànd convicted. The consequence of non-prosecution for those from outside the EU is that they likely will not be allowed to stay. Participants mentioned several reasons for not wanting to go home, including the unsafe situation back home, the lack of economic prospects there, and that they had become a little bit Dutch in the time they were in the Netherlands.

Some, particularly several men, expressed a sense of urgency in realizing their envisioned future: “I have not been having access to so many things. So I am looking at my future as being bleak. You know? So, where am I going? You know? And at my age now, the age I’m now, either you make it or you break it.”

#### Looking forward: thinking about the future and its effect on the present

Thinking about the future was not of immediate concern to most study participants when they had only just entered the shelter. Several stated that they first needed to ‘catch their breath’. However, there was a moment at which this changed for them. As one participant said: “The longer you stay here, well, the more your mood changes, so to speak. ( …) There comes a moment when your mood changes, when you start thinking, where do I go from here?” Some appeared to transition quickly to this new state of mind in which the future took up a more dominant part of their thoughts: “I could press charges immediately, but I was really afraid. I had to take my time and three days were enough to take a decision.” Others needed more time; one participant in particular stated at the time of her interview: “I don’t have any plans for the future yet; I need to catch my breath first.”

However, this participant was an exception; with all others their envisioned futures, and issues around those futures, took up a significant proportion of the interview. Interestingly, thinking about the future meant thinking about the past. As one participant said: “The most important thing is, ehm, I just want to get rid of my life, you know, living in places where I will forget about my past, you know, my past, just think about what the future holds for me, you know?” Forgetting about the past and moving towards a better future seemed almost inextricably linked concepts for interviewees. A particularly striking example of this link between the past and future was that one participant noted that two religious verses had particularly helped him to calm down in the shelter: one asking God for protection from evil men (“Contend, O Lord, with those who contend with me; fight against those who fight against me. Take up shield and armour; arise and come to my aid. Brandish spear and javelin against those who pursue me”); another asking God to help him be a vessel for change in the future (“Lord, make us a channel of thy peace. That where there is hatred we may bring love.”).

This need to ‘turn over a new leaf’ meant that in one move, participants envisioned themselves as leaving the past behind and moving to the next page of life. This theme, like most of the themes in our analysis, was suggested by one of the participants, who said about the interview that she was scheduled to have with the police the next day: “( …) it is a difficult issue to talk about for me. I do not want to talk about it at length. I do not want to go back in my memories. That was a difficult time in my life, which is why I would like to turn over a new leaf now.”

As noted earlier, that leaf had not been turned over yet for participants. Thinking about the future had both positive and negative consequences as described in the following two sections.

##### Positive effects of looking forward on the present: acquiring agency over goal pursuit

A salient characteristic of participants’ presents was that almost all exhibited signs of actively *working towards* a better future. This was expressed in terms of some of the activities that they reported appreciating. Dutch lessons, particularly, were appreciated by almost all, as were the integration courses offered by Dutch municipalities to people in asylum procedures. Participants felt these activities would help them establish their future in the Netherlands. Language was seen as a pre-requisite for integration, finding a job, getting an education and, in turn, establishing a family and a social network in the Netherlands. As one said: “This is a different country, (…) you have to learn the language, you have to mix with the culture, believe traditions, and all the rest of it.”

Skills training and the opportunity to volunteer were also broadly appreciated. As one participant explained: activities that you can learn from are good, “such as self-defence, where you learn to protect yourself and control a situation”. Another explained that she appreciated vocational skills training, because it kept her busy, gave her more passion about life and was good for her development, making her more independent. A third said: “I said I can look for work or volunteer work? I said that is ok. Because you cannot be depending on someone for your whole life. I need to do something by myself. I cannot say: give me something to eat, give me this/that … Someone must think: this is someone who can take care of herself. (…) Sometimes it’s good to be busy. To be busy with something good. Or with something better than some of the activities, with things that are necessary.”

What all these things (Dutch language training, skills training and volunteer work) have in common is that they allowed participants to learn and that they were *useful*: they helped them get one step closer to their envisioned futures. Volunteer work, for example, brought them one step closer to finding a job, an essential part of those futures. As one participant noted: “When I go out from here, living on my own, I need job also, so I need only that job from [name shelter] to help me.” Moreover, volunteer work gave participants a feeling they could contribute to “things that are necessary” – also a part of their envisioned futures. In other words, doing volunteer work, learning Dutch and acquiring skills allowed participants to ‘have a meaningful day’, by feeling competent and by giving them a sense of agency over pursuing their future goals.

‘Putting down roots’ was also an important theme in terms of looking forward. It indicated that participants were already starting to move towards their better future. There was more to this theme than settling in; over time participants anchored themselves more and more to their environments, both in terms of their relationships and the activities in which they were engaged. They started making friends both in- and outside the shelter: the latter happened via churches that they visited, but also via other groups, such as peer-support groups: “Imagine you meet people who speak the same language, eat the same food as home – I was so very happy that day.” Some participants had also grown attached to certain service providers, such as their psychologist. One noted, for example: “I felt good with this psychologist, my nightmares had stopped and I felt less stress. (…) With her it was possible to discuss your problems, and she understood and gave recommendations. That is sometimes enough, if someone just listens to you and understands.”

Putting down roots was a positive experience. This might have been because the ability to develop a network of friends and to establish familiarity with people who they interacted with allowed participants to develop a secure base for themselves. As migrants, this was a development of significant relevance to them, and as people who had been through a traumatic experience, arguably, even more so. While the activities mentioned (language lessons, skills training and volunteer work) were considered positive by participants because they might help them in attaining their future goals, putting down roots must have felt for them like they were already moving forward: step by step, the present and envisioned future were blending together.

However, this positive development had the potential to turn into a negative one. Particularly, the COSM shelters are a form of crisis shelter, intended to shelter people for a maximum of 3 months: the consequence of these long stays was that many participants started to put down roots. However, all at some point had to leave and the long-term shelter to which they then moved was often in an entirely different city. One participant’s second interview was conducted after she had moved to such a long-term shelter. She reported travelling back regularly to the city where she was originally sheltered to see her friends and noted that several others did the same. She said: “It’s very far from the people you know, from my church, from the activities I started.”

Several other participants also moved during the research, because one COSM shelter moved. Two of them described having developed a fruitful relationship with a psychologist in their first place of residence, which they were not able to continue after they moved. Thus, although putting down roots meant for participants that they could start moving forward, when they had to move, they were set back, not allowed to take off into their envisioned future, having to start anew again somewhere else.

##### Negative effects of looking forward on the present: the gap between reality and the envisioned future

For most, there was a large gap between their present realities and their envisioned futures, a sign of the ‘in-between state’ they were in. This gap became very clear from the fact that the future for all study participants was characterized by great uncertainty. “Do you think I have a future?” one asked at some point. Later she said: “You will never know your future here”. Similarly, another described: “Look at my situation, yeah. Where am I going to? Where is my future? What will happen to me?” There were several reasons for uncertainty about the future: doubts about residency status were a common concern; as were doubts about being able to find work or an education and to establish a family and make friends; the legal case against the trafficker; and for some, uncertainty about the future was linked to the whereabouts of loved ones.

All this uncertainty led participants to worry. One explained, for example, that while she had trouble forgetting about the past, her worries about the future were particularly hard to deal with: “First, I have no residency status here. I’m afraid they’re going to send me back. Besides that, I have no education or work experience, so that’s why I’m worried.” These worries about the future were a source of stress that added to the stress associated with dealing with the past. When participants spoke of ‘being busy’ as a form of distraction from their problems, they did not speak only about distraction from thinking about the past, but also from worrying about the future. As one noted: “Everything is sometimes stupid, you just have to be quiet sometimes, and think about the future, and the past and everything in your life. That is not going to be happy.” Another described well how these various stressors could accumulate: “Sometimes it’s just too much. I thought: Why? Why me? One thing come, and then the other.”

A key facet of participants’ feelings of uncertainty was the lack of autonomy that they experienced. Indeed, in many decisions of importance to their futures, the locus of control did not lie with participants themselves, and they experienced a great sense of dependency on the police, lawyers and social and health providers. Examples of such dependency for participants while they resided in the COSM shelters were: that one participant had her child taken away from her by the Dutch child services; that residency status was decided upon by the police and by lawyers; that procedures around human trafficking are complicated and often ill-understood; and that most service users were legally not allowed to look for work and education. One participant remarked, for example: “You’re the one who needs help, so you’re the one who needs to accept everything”. And: “I am here but I don’t know what is going on. They will never tell you what will happen. You’re just here and never know what is next.” And: “Life just happens to me”. A striking demonstration of the lack of power that participants had over their own lives occurred when they received a letter that the police did not have enough evidence to prosecute the trafficker, meaning they would likely not be allowed to stay in the Netherlands. This was devastating to many.

In some instances, the shelters contributed to the lack of experienced autonomy. The shelters had rules and regulations by which service users had to abide. Examples were: mandatory activities, curfews, mandatory visits to psychologists, not being allowed to have a phone, having no choice in the assignment of roommates, not having ownership over one’s own government benefits, and being asked upon arrival at the shelter to sign a form (in Dutch) agreeing with the shelter rules. Participants spoke in particular about their objection to not being allowed to have phones and the mandatory assignment of roommates. One interviewee continued to clash with service providers over these rules. She said: “My social worker forced me to sign a protocol. And if I break the protocol three times I am removed from the house. I told her: You can make five more protocols, but this is the worst house I have ever lived in.” In the end she decided to leave the shelter. The number of rules and regulations differed considerably by shelter; one in particular had more rules than the other two.

Another part of the gap participants experienced between reality and their envisioned futures was that they felt ostracized in the Netherlands; they still had to adapt to the language, the culture and the traditions. As belonging in Dutch society and building a life there were part of their envisioned future, their experienced difficulties in this regard were particularly salient to them. Lack of integration was demonstrated most extremely by the fact that several participants told of instances when they were met with hostility and racism. Such instances appeared to occur more frequently for those who were sheltered rurally than in a large city. With one rural shelter in particular, there were several examples of service users being discriminated against, including being denied parts of their benefits to which they were legally entitled by the city council and being denied care by a general practitioner and a dentist. Several also encountered hostilities from local residents, such as being bullied by local teens and being maltreated or ignored by adults. Participants found being treated as such dehumanizing: “Imagine the situation wherein, you know, some people don’t want to talk to you, then you feel you are left out, you feel maybe I’m not in this world, or maybe I’m a different human being, you know?” The opposite of such dehumanizing treatments was found by some participants to lie in their interaction with service providers in the shelters. As one explained: “They refer to us as maybe say we are ‘asielzoeker’, which means something like refugee, something like that. But because of some [service providers], they come closer to us, we feel as if we are amongst them. (…) You know, so we feel as if really we are part of this society.” This particular sentiment appeared to be part of what participants more broadly considered being the most important aspect of a good service provider: somebody who shows interest, listens, understands and gives advice when needed.

Thus, while study participants desired strongly to move towards a better future, they lacked agency in bringing about that future, resulting in uncertainty about the future and leaving them thwarted in their goal pursuit. The rules and regulations in the shelters added to participants’ lack of experienced agency in moving forward and their ostracized place in Dutch society increased the gap that they experienced between their realities and their envisioned futures. The result of all this was that participants felt as if they were ‘standing still’ while wanting to move forward, as in a state of limbo. This feeling stemmed partly from the gap between reality and their envisioned futures, but mostly from the gap between wanting to work towards better futures (pursuing their goals) and being thwarted in doing so. One participant said, for example: “I’m seeing my days here as something like, what can I say, perhaps I should say it’s my waste days, you know, like I’m wasting my time here.” Another perhaps made clear best the degree of ‘standing still’ that they experienced, by explaining about a trip to the general practitioner: “If I have an appointment with my doctor for, for … I mean that would be my happiest moment. Because, the reason, I look at it that I need to get up early, I need to go down to get bus station, go get bus, you know, that time that we spend in the bus, I will see different people, then the time it will take to go to […], I will see different places, then when I reach […] again, before my appointment with the doctor, I need to see different people, you know? You know, that different environment alone that I’ve seen, you know, that create a new, different thing in my mind.” Standing still was not only a negative experience in itself, but also resulted in participants not ‘being busy’, resulting in turn in a lack of distraction from past problems and future worries. As the same participant explained: “But sitting here all day, you know, since morning evening, morning evening, morning eve – you don’t get any other places you just sitting down in one particular place, it’s very, very, very difficult, in fact that one make the situation more difficult than the situation is.”

## Discussion

Participants’ self-representations and self-evaluations in these three post-trafficking shelters commonly showed a clear temporal pattern. People who experience rapid change, particularly when the affective quality of the present is negative, are more likely to draw temporal comparisons, potentially explaining why this theme was so dominant in participants’ narratives [47]. The temporal pattern had several characteristics: the past was generally evaluated negatively, deemed as something to forget and not to return to; the present was an ‘in between state’; and the future was envisioned positively, as a life with a job, family and friends. This pattern matches the ‘progressive pattern’ in Gergen’s theories of temporal self-evaluation [43]. Looking forward, and being able to work towards a better future, gave meaning to participants’ presents. However, in the pursuit of those goals, they were often thwarted, because many decisions that were made about their lives were outside the locus of their control. This left them in a state of limbo; not quite in the past, yet not quite in the future either, and being prevented from pursuing their goals in the present. As a result, participants experienced uncertainty about the future. Both thinking about the past and worrying about the future were causes of stress for them in the present. Despite this, they found ways to work towards a better future, for example through language classes, skills training and volunteer work.

Here we interpret these findings using theories of self-determination and hope, further exploring the notion of being ‘in limbo’ and specifically the concept of bounded agency and how participants were able to pursue goals, seemingly against all odds. Finally, we also discuss the implications of the findings for service provision.

### Self-determination and hope

Participants’ envisioned futures consisted of a life with a job, family and friends. Integral to these aspects of their future lives was a desire for achieving autonomy. These findings are not unique to this population. When other marginalized groups, such as the homeless, are asked, they also indicate the lack of fulfilment in these areas to be most salient [48]. The apparent broader relevance of these findings can be explained through the theory of self-determination, which postulates that all human beings have three basic psychological needs: competence, relatedness and autonomy [49].

The need for *competence* (“individuals’ inherent desire to feel effective in interacting with the environment” [50] in order to reach their goals in life) by participants in this research became clear from their future goals to find education and a job. They also strived for competence in the present. Often these short-term competence goals enabled them to pursue the longer-term goals of work, family and friends. Examples of such short-term goals were learning Dutch, doing well in integration classes or gaining other practical, potentially beneficial skills. But competence in the short term was not always driven by long-term goals: some participants indicated deriving satisfaction from having done volunteer work simply because it mattered (as well as providing distraction from their problems and worries).

Participants’ need for *relatedness* (“individuals’ inherent propensity to feel connected to others” [50]) showed from their future goals of having a family and friends and the need to belong in Dutch society and gain a safe basis for the further development of their lives. This need was not independent of the need for competence, which was viewed by several as a precondition for having a family. As with competence, participants also exhibited a need for relatedness in the present, as was clear from the signs of putting down roots, the joyous descriptions of connecting with people from the same country, and the negative descriptions of being ostracized in Dutch society.

The need for *autonomy* (“individuals’ inherent desire to feel volitional and to experience a sense of choice and psychological freedom when carrying out an activity” [50]) was a key theme throughout participants’ descriptions of their envisioned futures. Examples in the present were the pride that one participant took in opening a bank account and the negative experiences of autonomy-limiting rules in the shelters, such as not being allowed to have a phone. Deci and Ryan, who laid the foundations for self-determination theory, write that “autonomy occupies a unique position in the set of three needs: (…) being able to satisfy the need for autonomy is essential for the goal-directed behaviour to be self-determined and for many of the optimal outcomes associated with self-determination to accrue [49].”

Self-determination theory provides an explanation for the predominance with which themes such as work, family, friends and the wish to belong in society emerge from this study. The theory explains why people strive towards these goals in general: the fulfilment of the three basic needs has been shown to be associated with increased well-being [51]. However the theory may apply particularly to this population, because of the traumas many have experienced. Research shows the fulfilment of these three basic needs is not only associated with increased well-being, but also with better *coping* [51].

Lazarus distinguishes two types of coping: emotion-focused and problem-focused coping [52]: “When stressful conditions are viewed by a person as refractory to change, emotion-focused coping predominates; when they are appraised as controllable by action, problem-focused coping predominates” [52]. This may explain why several interviewees spoke of ‘turning over a new leaf’. Pursuing future goals allowed for a problem-focused approach to coping, since it was linked with leaving the negative experiences of the past behind. This suggests the pursuit, not just fulfilment, of the three basic psychological needs is associated with better coping, as Deci and Ryan also argue, noting the importance of both goal *pursuit* and *attainment* in predicting behavioural quality and mental health [49].

Related to the concepts of goal pursuit and goal attainment is the concept of *hope*. Hope for the attainment of goals is an integral part of goal pursuit, but this is not necessarily the case vice versa. Snyder et al. define hope as “the perceived capacity to produce pathways to desired goals (pathways thinking), along with the motivation to begin and continue the use of those pathways (agency thinking)” [53]. Similarly, Averill posited that hope can only occur when the goals are also “under one’s control” [54]. In other words, hope is the wish for a possible goal under the conditions that one is motivated to work towards that goal and that one considers autonomous pursuit of that goal possible. Thus, hope does not necessarily involve goal pursuit (only the possibility thereof), while goal pursuit always involves hope. Hence, hoping for goal attainment is one step further removed from actual goal attainment than the act of goal pursuit (and wishing yet again one step further away).

It has been shown that even merely *hoping* for the fulfilment of the three basic needs is associated with better coping [55]. This has interesting consequences when viewed in light of our findings. However, since hope is strongly linked to agency over goal attainment, to discuss these consequences it is necessary to first discuss the agency over goal attainment experienced by participants.

### In limbo

The salient characteristic of participants’ presents was that they felt they were ‘standing still’, in a state of limbo. A lack of agency in changing that situation was experienced. There were many external regulators that forced participants to accept decisions about their lives that lay outside their control and sometimes even outside their knowledge. The most prominent expression of this was the dependent position they occupied in the migration system. For study participants from outside the EU, migration to the Netherlands was considered to be a precondition for a better future. The decision-making mechanisms for a residency permit were outside their control and many participants were not knowledgeable about the procedures involved.

A variety of policies, rules and regulations at the shelters also contributed to this experienced lack of agency because they blocked participants from being autonomous. Brunovskis and Surtees have written in detail about the restrictions regularly put on trafficked people in shelters. They conclude: “Restrictions may infantilize program beneficiaries and impact their agency and ability to dissent and negotiate within the program framework.” [56] A further complicating factor was that most participants felt there were few possibilities for leaving the shelter. Administrative procedures often meant that they could not apply for follow-up housing for several months and the lack of a social network in the Netherlands limited their options for seeking alternative housing*.*

**Fig. 1:**
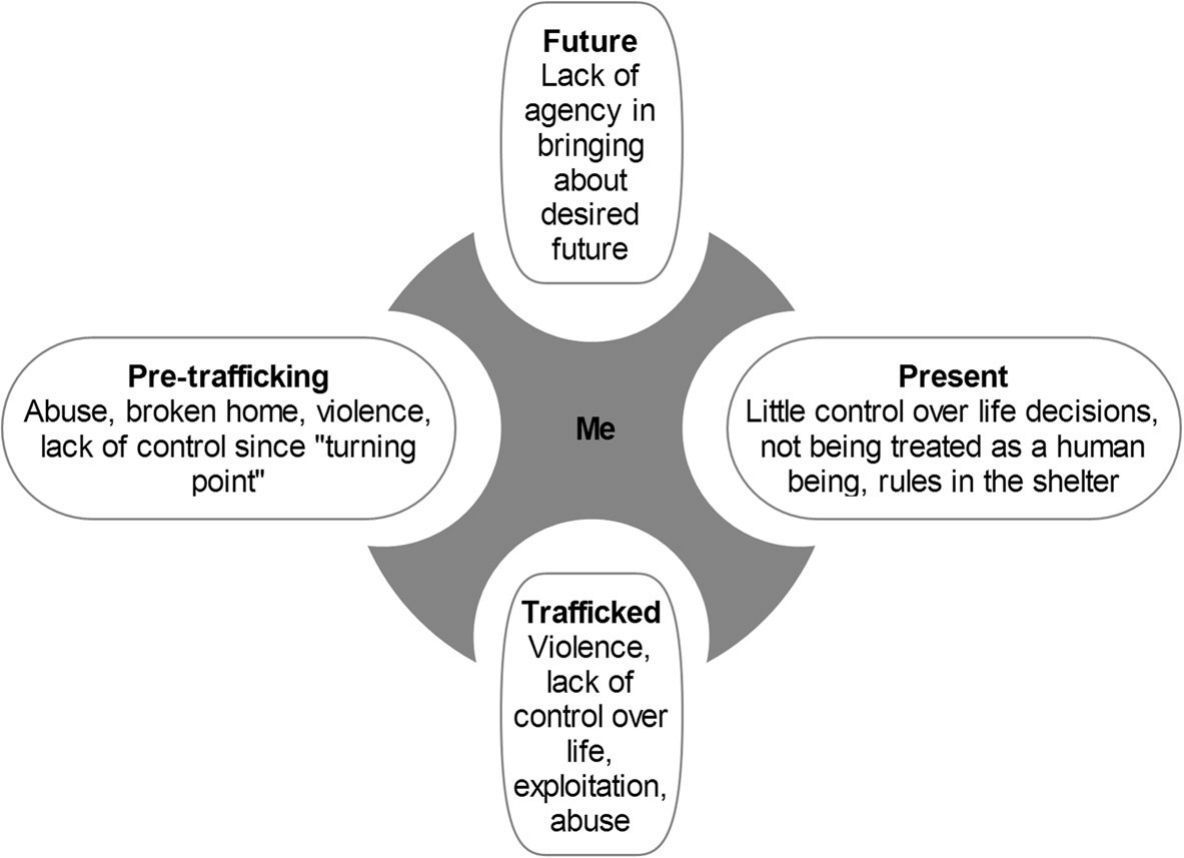
Loss of autonomy in various phases of the lives of trafficked persons. The figure shows the various phases and examples of external negative regulators that give rise to experienced loss of autonomy

In a way, participants were bound to the migration system and the COSM shelter system and had little power to negotiate the conditions of either. Their ostracized place in Dutch society and the general lack of relatedness to people outside the shelter confined them even more. Participants’ lack of experienced agency in changing their situation because of a lack of autonomy was not new for most. Lack of autonomy is a key aspect of trafficking experiences and many experienced a ‘turning point’ even before their trafficking experiences that signalled the beginning of an era of non-autonomy. Lacking the freedom to make decisions over one’s own life at various stages in the past and present (and about the future) (Fig. 1), it is, perhaps, no wonder that participants felt trapped in the events that befell them. As one said, “life just happens to me”. Others also refer to the recurring nature of external negative regulators for trafficked persons, for example, Wilson, who speaks of them running “a gauntlet of victimization and violence” [57].

The consequences that experiencing such a lack of agency in goal attainment can have on people are well explained by self-determination theory, which states that the strong presence of external regulators prevents self-determined behaviour and stunts the intrinsic motivation towards goal pursuit [49]. Deci and Ryan write that when “in spite of people’s persistent attempts to satisfy the fundamental needs for competence, relatedness, and autonomy, if the social world provides no reliable paths that allow fulfilment of these critical needs, and if people have to stay in situations that consistently block need satisfaction (e.g., children often have to stay in non-nurturing homes and schools), self-determination theory predicts significant psychological costs and accommodations” [49]. Participants’ remarks that they could not “recognize themselves” might be an expression of these detrimental consequences.

Parallel to the lack of experience of agency in goal attainment, participants also showed a strong drive to develop competences that could help them achieve their future goal of a life in the Netherlands with a job, a family and friends, such as learning Dutch, acquiring vocational skills and engaging in volunteer work. This presents us with a conundrum: how could participants exercise agency in goal pursuit, while they simultaneously experienced little agency over goal attainment?

The answer to this question might be found in theories about self-deception. Self-deception in the face of uncertainty about the future is not only common, but it is human [58]. Robinson writes that “in perceiving the future self, most of us appear to be great optimists, overestimating the likelihood that positive events will happen to us as well as underestimating the likelihood that negative events will happen to us.” [58] Having overly positive illusions about the attainability of goals provides us with a sense of agency over goal attainment. It offers the possibility of goal pursuit and thus allows us to hope. We are all wired, it seems, to hope for the best.

Yet, participants’ accounts did not show any signs of such self-deception. Participants did not display what Snyder describes as being “overly positive” [55] or Robinson’s description of overestimations of likely outcomes of positive events [58]. They were distinctly aware that many decisions lay outside their control and that those decisions may result in non-attainment of their goals. Despite realizing this, they established pathways toward goal attainment (e.g. learning Dutch) and actively pursued their goals, even though a decision on migration status might stop any chance of a future in the Netherlands. It is not so much that they deceived themselves into believing that the external loci of control did not exist; but that despite of the existence of these external loci of control they chose to focus on the pathways that they *could* control. This behaviour can be explained through theories on the concept of bounded agency [59]. Evans, in explaining this concept, states that the concept sees actors as “having a past and imagined future possibilities, which guide and shape actions in the present, together with subjective perceptions of the structures they have to negotiate, the social landscapes which affect how they act.” [59] The fact that participants still showed agency in pursuing their future goals, despite being bound to the migration system and the COSM shelter system, with little power to negotiate the conditions of either, provides testament to the fact that even in a context with highly binding external regulators, people will continue to exercise agency to pursue their goals; in a way, agency against all odds.

This leaves the question of *why* participants were so driven in their goal pursuit. The answer to that question can be found in the effects of hope and goal pursuit, discussed above. By focusing on the pathways that they could control, participants enabled themselves to hope for goal attainment (remember that hope requires both a pathway and agency over that pathway – the possibility for goal pursuit) and to actually pursue their goals in the context of highly binding external regulation. Both hope for goal attainment and goal pursuit are associated with better coping and well-being. This suggests that by fighting the fights that can be fought, participants gave themselves a fighting chance.

### Implications for service provision

The main lesson that follows from this study is that to facilitate service users’ recovery as part of post-trafficking shelter and care, there is a need to provide them with opportunities to hope for, pursue and attain their personal goals within the structural boundaries of their situation.

#### Lessons for service provision approaches

Existing frameworks for the post-trafficking social and health care needs of trafficked persons generally adhere to a model that consists of a thematic categorization of different social and health care needs, sometimes differentiated to two or three different stages of recovery (Table 1) [3].

**Table 1:** Needs for services in a Dutch shelter for adults of non-Dutch nationality who were trafficked and descriptions of those services

Services that are needed in a shelter for adults of non-Dutch nationality who were trafficked	Descriptions of services
Physical health care	Care that is provided when trafficked people experience physical health problems. This care can be provided by social care workers in the shelter or by a GP that is affiliated with the shelter. Trafficked people may be offered screening for sexually transmitted infections (STIs) and tuberculosis.
Mental health care	Care that is provided when trafficked people experience mental health problems. This care can be provided by social care workers in the shelter. Additionally, mental health care may be offered via mental health care institutions.
Safety	This service consists of advice to trafficked persons on how to increase safety, ensuring security at the shelter itself, accompanying them to places if necessary, and assisting them in contact with the police if necessary.
Emergency shelter	Providing shelter is the main function of a shelter, offering sleeping quarters and communal areas that are arranged in varying manners.
Longer-term housing	This service consists of helping trafficked persons with looking for a different place to live after they have to leave the shelter and wish to remain in the country where the shelter is located.
Legal assistance	Trafficked people should receive legal assistance. This may be offered in the form of basic advice by social care workers in the shelter. They may also be referred to a counsellor for more advanced legal assistance. Assistance may include help with acquiring residency status, representation of the service user in the trafficker’s criminal case, and other matters such as acquiring financial compensation from the government.
Translation	Translation services should be freely available to the shelter, financed by the government.
Daytime activities	A range of day activities may be offered at the shelter. The nature of activities may differ by shelter, but can include cultural orientation, language training, occupational skills training and arts classes.
Help with education / employment	This may differ by shelter but can include occupational skills training, advice on possibilities for (voluntary) employment during stay at the shelter, help with finding education, help with finding longer-term employment, and referral to organizations that assist people in finding employment.
Financial and administrative assistance	Services may include help with getting subsidies and social benefits, help with filling out papers and forms, advice on how to get by with little money, and help with debts.
Empowerment	Key to services that aim to empower people is that they are founded on people’s strengths. The central goal of empowerment is that trafficked persons (re) gain control over their own life and their surroundings, so that full participation in society can be achieved.
Self-maintenance and -care	Helping people take care of themselves. Services can include help with cooking, grocery shopping, and laundry.
Child care	Services may differ by shelter but can include helping people with taking care of their children, advising on possibilities for helping children, helping to establish contact with relevant agencies, and contacting the relevant agencies when there are worries about the parent(s) to be able to care for their child.
Social contacts	Help with establishing rewarding and fruitful social contacts (including family and friends but also others such as care providers or colleagues). Services may include advice on ways to get to know other people, advice on discussing problems with people, learning how to say no, and learning how to ask for support.

This framework was developed by combining frameworks from international research on the service needs of trafficked persons with frameworks for service provision by Dutch shelters to comparable groups [1, 3, 60, 61, 78–80]

These frameworks are useful for helping service providers to consider the different kinds of services that need to be in place. However, the frameworks exhibit a strong focus on practical needs, protection and helping people to deal with traumatic events of the past. While these are all indispensable components of the broad spectrum of social and health services that need to be available for this group, the relatively minor attention that service users’ goals for the future receive in these frameworks is in discordance with the importance attributed to hope and goal pursuit by participants in this study. Some frameworks do mention one or two service needs that relate to the three basic psychological needs listed in self-determination theory, such as job training and employment, the development of a social network or long-term housing [60, 61], and so do many individual papers, such as a paper by Busch- Armendariz et al. about reunification of people who were trafficked across borders with their children [32]. Yet, commonly, these needs are described as “long-term needs”, suggesting that service users first need to stabilize [61]. While stabilization certainly should be the priority in the short-term, the findings of this research suggest that service users start thinking about their future goals after a short period of time. They also suggest that working towards a future can help service users cope with events in their pasts – allowing them to ‘turn over a new leaf’ using a problem-focused approach to coping – and that if the need to look forward is not accommodated, service users will feel themselves to be standing still in a state of limbo, with potential negative consequences for their recovery. Moreover, Snyder et al. have shown that people may need help with goal-setting, pathway development and developing agency over goal attainment [55]. Therefore, a more prominent focus on creating opportunities for service users to pursue the goals of competence, relatedness and autonomy would likely make a valuable addition to the existing social and health care provision frameworks for trafficked persons. We propose that this be added to the recommended practices proposed by others for service provision for this group (if the framework in Table 1 is ‘what’ should be provided, these best practices describe ‘how’ services should be provided). Practices recommended by others in the past are that social and health services for trafficked persons should: (see chapter 2 in [3])

- be comprehensive
- be integrated and/or coordinated
- allow for continuity of service provision
- be population-specific (“categorical”)
- provide individually tailored care
- provide culturally appropriate care
- be trauma-informed
- offer case management

This article shows that another best practice should be added to this list:

- be future-orientated

The next question is then how a more prominent focus on service users’ goals for the future might be established. For this, lessons can be learned from other vulnerable populations. Trafficked persons have been conceptualized as a population that shares characteristics and service needs with several other vulnerable groups [1, 62, 63]. In this study, participants displayed both perspectives found amongst migrant groups and perspectives shown to be held by people who have been subjected to violence [1], as became clear from the difficulties that many reported experiencing in relation to their residency status and the difficulties that some experienced in dealing with their violent pasts. Therefore, looking to lessons learned from service evaluations for these groups may provide useful insights for service provision for trafficked persons.

When discussing such research about care for people subjected to violence in the Netherlands, it is important to note that there has been a recent shift in social care policies for this group. For years, the dominant model of guidance for social service provision was the ‘8-phase model’; a staged approach to service provision, including a thematic categorization of different social and health care needs. Recently, after research by Wolf on the social care practices for people who have been subjected to domestic violence and homeless people in the Netherlands, this model was replaced by a working methodology called ‘Strength-work’ [64], which is based on the ‘Strengths model’ [65, 66]. This working methodology has a strong focus on helping service users establish a safe and meaningful life towards full citizenship and on helping people develop agency in working towards that future, by taking their strengths (instead of their problems) and personal long-term recovery goals (derived from a strengths assessment) as the starting point for service provision. This approach aligns with the future-orientated views participants displayed in this study. As one noted: “I want to start taking care of myself; I don’t always want to be a social case. I have capacities too! (...) It’s just, I have no anchor point currently, which is why I can’t do it.”

When looking at social and health services research in the Netherlands for asylum seekers, two recent studies in particular bear relevance to the findings of this study. The first, aptly titled “Small steps of great meaning”, investigated shelter and care provision for asylum seekers and recommends a more humane policy in service provision for this group [67]. It too speaks of an in-between state that service users are in, discusses the importance of agency in goal attainment and concludes that “only when people are not limited to one aspect of their identity, that is being an asylum seeker, but when dreams, ambitions and talents are given the opportunity to develop, can we speak of a truly humane approach to care” [67]. The second, a report of minors seeking asylum in the Netherlands, called “Waiting for your future”, speaks of the uncertainty that children are faced with and the difficulties they experience in “dreaming about a future, let alone shaping that future” [68]. The findings of these reports show striking resemblances to our findings and provide important confirmation of these findings from a group that is partially comparable in terms of service needs. Moreover, this also suggests that while the health consequences of violence associated with human trafficking have received a lot of attention in the literature [46, 69], the migrant narratives of trafficked people may not have received enough attention. A greater focus on this part of their service needs may help to achieve a more future-orientated approach to service provision for trafficked people. The Belgian non-governmental organization Flemish Refugee Action has recently proposed a model for what a future-orientated service provision approach for asylum seekers should look like [70]. In 2014, the Dutch Shelter Federation also acknowledged that there was a need for specialized guidance for trafficked people of foreign origin in shelters [71, 72]. This “Safe Future” approach builds from approaches in service provision for asylum seekers and refugees, such as the Flemish Refugee Action model [70], and from the Strength-work methodology [64–66].

Finally, in considering how a more prominent focus on service users’ goals for the future might be established in social and health service provision, it is notable that self-determination theory speaks extensively about the importance of enabling environments in needs satisfaction [49, 51]. Specifically, it reports that responsive and supportive contexts, by improving resilience, well-being and capacity for coping, are vital to enable people to overcome situations in which their basic psychological needs are frustrated [73]. Trafficked people will likely benefit from service provision approaches that adopt these values and that also promote broader social environments that enable them in this way.

#### Lessons for the research context (the COSM programme)

In addition to lessons for service provision approaches, several lessons follow from this study for the Dutch COSM programme. First, it is now broadly accepted that a specialized approach to service provision for trafficked people is necessary, given their complex array of problems [7, 74]. Yet, the Dutch COSM programme started as a ‘bed-bath-bread’ programme (a Dutch term), financed only to provide merely the most basic services (it now includes broader service provision).

Second, as noted above, self-determination theory emphasizes the importance of an enabling environment to achieve needs satisfaction. Autonomy support, structure and interpersonal involvement provide such an environment and support need satisfaction [51]. There are various ways in which these can be achieved in service provision. Direct service provision practices, the shelters’ vision and approach to service provision, and the availability of additional services outside the shelter all play a role. The previous section makes clear that lessons might be learned from various service provision models in considering these aspects of service provision [64–66, 70]. Hence, it deserves recommendation that the COSM shelters evaluate the degree to which their service provision is in accordance with the best practices advised by these approaches.

One example of a lesson that the COSM shelters might derive from these approaches relates to the shelters’ overall visions. The three COSM shelters that participated in this research had strongly opposing visions for service provision: one took a structured approach, with many rules and daily schedules, proposing that structure promotes activation and, as a consequence, recovery; while the other two shelters took a personal freedom approach, with few rules and no daily schedules or activities, proposing that personal freedom and regaining agency were key to recovery. When assessed against self-determination theory’s recommendation for autonomy support, structure and interpersonal involvement, both approaches have their strengths and weaknesses. Many participants in the first shelter felt controlled and experienced a lack of autonomy, which even caused one participant to leave. However, participants in the shelters with no activities felt that they were ‘not busy’, which also had detrimental effects on their recovery. Ntoumanis et al. suggest that the optimal approach might lie somewhere in the middle. Structure and interpersonal involvement are important, yet the manner in which these are provided is crucial – in a way that supports service users’ autonomy [51].

Another example concerns the shelters’ direct service provision practices, in particular the daytime activities. This study has shown that for trafficked people in a shelter, these activities are much more appealing when they are ‘useful’, that is when they help them pursue their goals. More emphasis should therefore be placed on the provision of such activities, rather than those that are mainly aimed at distraction.

Lastly, participants’ experiences of standing still and being in limbo were exacerbated by a number of challenges in the service provision system. These challenges, and what might be done to redress them, are described elsewhere [3]. This research makes clear how important it was for participantsto be able to hope for and pursue their own personal goals. The fact that participants were thwarted in doing so, partially owing to existing challenges in the service provision system, demonstrates the significance and impact of those challenges and the importance of redressing them.

### A note about differences between men and women

Sexual exploitation of men is an under-recognized problem, perhaps because it is such a taboo. Some describe it as a triple taboo: a taboo on doing sex work, a taboo on homosexuality and a taboo on male victimization [75]. Yet, many reports show that it does take place with significant frequency [6, 75, 76]. Few differences between men and women were encountered by us in the problems they faced, in how they envisioned their futures, and in how they experienced service provision. This makes clear that trafficked men must be included in debates about service provision after human trafficking for the purpose of sexual exploitation and should be viewed to have social and health care needs in this regard. The specific needs of trafficked men versus trafficked women merits further research.

### Limitations

The research conducted for this chapter has several limitations.

The first limitation is that participants were interviewed at least 6 weeks after their first entry to the COSM shelter, with a median of 2.8 months (this was a safety measure). As a result, the findings of this chapter, including my analysis of participants’ goals for their own recovery and of their experiences with service provision, apply particularly to the later stages of trafficked people’s stays in the shelters. In the earlier stages, safety and stability have been shown to take up a more prominent place in trafficked persons’ needs profiles [77].

It is also important to stress other limitations of this research’s study population. This study only included trafficked people who received post-trafficking shelter and care, which is only a small percentage of all trafficked people [6]. Moreover, this research was limited to adults and to people of non-Dutch nationality, and to those who were trafficked for the purpose of sexual exploitation. The transferability of this research beyond this population is limited.

Another limitation is that this research was conducted solely in the three COSM shelters, a setting with a specific programmatic, social, medical and legal context. Therefore, the findings of this chapter only bear direct relevance to this population and this context. However, that said, Green and Thorogood have argued that the overarching themes that emerge from qualitative research are often more transferable than studies’ context-specific findings [21]. By linking these overarching themes, which consisted of descriptions of participants’ goals for their recovery process and their experiences with that process, with theories of hope and goal pursuit in the Discussion, we have tried to further increase the transferability of the findings. Doing so, we hope to have argued that the strong drive for goal pursuit experienced by foreign trafficked persons can be expected to exist in other contexts as well.

## Conclusion

This study shows that its participants – people who were trafficked and now receive shelter and care in the Dutch ‘COSM programme’ – exhibited progressive temporal patterns of self-representation and self-evaluation. They strongly wanted to fulfil the basic psychological needs of competence, relatedness and autonomy, but were thwarted in pursuing a safe and meaningful life with a job, a family and friends. Finding ways to pursue those goals nonetheless, regardless of the challenges that existed in doing so, allowed service users to hope for a better future. Both pursuing their goals and hoping for their attainment helped service users to cope with the problems of their past and their worries about the future and increased their well-being. To facilitate service users’ recovery in a post-trafficking setting, there is a need to provide them with opportunities to hope for, pursue and attain their personal goals within the structural boundaries of their present situation. Future-orientated, strengths-based approaches may help social and health services in that setting to do so.

## Additional files

Additional file 1: Layperson summary, policy brief and extended abstract. This additional file contains the layperson summary, policy brief and extended abstract belonging to this article. (DOCX 17 kb)

Additional file 2: Context: the COSM programme. This additional file provides additional information about the research context, the Dutch COSM programme. (DOCX 22 kb)

Additional file 3: Context: Topic guides for interviews with service users. This additional file contains the topic guides used for interviewing participants in this research. (DOCX 21 kb)

Additional file 4: Context: Extended description of data analysis methods. This additional file describes in more detail than in the main text the methods used for data analysis in this study. (DOCX 25 kb)

## Abbreviations

CoMensha: Dutch National Coordinating Centre against Human Trafficking
COSM: Categorical Shelter for People who have been Trafficked
