# Supplementary material for: The recovery experience of people who were sex trafficked: the thwarted journey towards goal pursuit
Source: BMC Int Health Hum Rights. 2019 Jan 22;19:3. doi: 10.1186/s12914-019-0185-7 (PMC6341539; doi:10.1186/s12914-019-0185-7)
Supplement: Supplementary file 1 — Layperson summary, policy brief and extended abstract. This additional file contains the layperson summary, policy brief and extended abstract belonging to this article. (DOCX 17 kb) [file 12914_2019_185_MOESM1_ESM.docx]

## Layperson summary

**This is a study of how victims of human trafficking experienced social and health service provision in a shelter specifically for this group in the Netherlands and how they viewed their own recovery. We interviewed 14 service users, who were all adults, of non-Dutch nationality and trafficked for the purpose of sexual exploitation. Nine women and five men were interviewed. Our results indicate that service users felt a strong need to turn over a new leaf in life, leaving the negative experiences of the past behind and moving towards a life with a job, a family and friends. However, they had difficulties in working towards these goals, because they did not have control over many decisions relevant to their lives. Together with the fact that service users were not a part of Dutch society, this led service users to feel like they were ‘standing still’ during their time in the shelter. This feeling of standing still resulted in service users finding it more difficult to deal with problems related to their pasts and worries about the future. Despite the barriers posed to them in pursing their goals for the future (a job, a family and friends), service users found ways to pursue these goals, by engaging actively in Dutch language training, vocational skills training and opportunities for volunteer work. This allowed service users to hope for a better future and helped them to cope with the problems of their past and their worries about the future, thus increasing their well-being. The main recommendation of this research is therefore that there is a need to provide victims of trafficking in post-trafficking shelters or care with opportunities to hope for, pursue and attain their personal goals within the limitations of their situation.**

## Policy brief

**This study investigates how victims of human trafficking experienced social and health service provision in a shelter specifically for this group in the Netherlands and how they viewed their own recovery. The study’s main conclusions are:**

- **Service users felt a strong need to turn over a new leaf in life, leaving the negative experiences of the past behind and moving towards a life with a job, a family and friends.**
- **They had difficulties in working towards these goals, because they did not have control over many decisions relevant to their lives.**
- **Service users also felt ostracized in Dutch society.**
- **As a result, service users found it more difficult to deal with problems related to their pasts and worries about the future.**
- **Therefore, is a need to provide victims of trafficking in post-trafficking shelters or care with opportunities to hope for, pursue and attain their personal goals within the limitations of their situation.**
- **Approaches to service provision that are future-orientated and strengths-based may help to do this.**
- **Responsive and supportive environments, both in the shelter and in broader society, are equally important in enabling victims’ recovery.**

## Extended abstract

Introduction

**In the Netherlands, around 200 victims of human trafficking seek social and health services from a shelter every year. In 2010, a new national shelter programme for victims of human trafficking was established to provide specialized shelter services for this population: the Categorical Care for Victims of Human Trafficking (COSM) programme. This article presents the findings of a study of how service users in the COSM shelters conceptualize and experience their own process of recovery.**

Methods

**Fourteen service users were interviewed at all three shelters of the COSM programme in the Netherlands. The study population consisted of foreign, adult female and male victims of human trafficking to the Netherlands, trafficked for the purpose of sexual exploitation. A grounded theory approach was taken to data analysis.**

Results

**Service users felt a strong need to turn over a new leaf in life, leaving the negative experiences of the past behind and moving towards a life with a job, a family and friends. In contrast with their willingness to work towards realizing such a future, they experienced a lack of autonomy, resulting in feelings of uncertainty about the future and a thwarted sense of agency in redressing their present situation. Together with the ostracized nature of their place in Dutch society this left service users in a state of limbo, where they felt they were standing still (while wanting to move forward). This feeling of standing still led service users to find it more difficult to deal with problems related to their pasts and worries about the future. They particularly appreciated Dutch language training, vocational skills training and opportunities for volunteer work, all of which helped them feel that they were getting one step closer to their envisioned futures.**

Discussion

**Service users exhibited a strong desire to fulfil the basic psychological needs of autonomy, competence and relatedness, but were thwarted in pursuing a life with a job, a family and friends. Seemingly against all odds, when faced with several external regulators that limited their agency to change their situation, service users found ways to pursue these goals, through their enthusiasm for activities that helped them get closer to their envisioned futures. Identifying pathways toward attaining their goals allowed them to hope for a better future. That hope and pursuing their goals helped them to cope with the problems of their past and their worries about the future. To facilitate service users’ recovery in a post-trafficking setting, there is a need to provide them with opportunities to hope for, pursue and attain their personal goals within the structural boundaries of their situation. A future-orientated, strengths-based approach towards service provision and responsive and supportive environments may help to do this.**
