# Supplementary material for: The recovery experience of people who were sex trafficked: the thwarted journey towards goal pursuit
Source: BMC Int Health Hum Rights. 2019 Jan 22;19:3. doi: 10.1186/s12914-019-0185-7 (PMC6341539; doi:10.1186/s12914-019-0185-7)
Supplement: Supplementary file 2 — Context: the COSM programme. This additional file provides additional information about the research context, the Dutch COSM programme. (DOCX 22 kb) [file 12914_2019_185_MOESM2_ESM.docx]

### Context: the COSM programme

Previously, shelter and care was provided for victims of trafficking in the Netherlands in shelters for victims of domestic violence, the homeless or migrants.^1,2^ In recent years, recognition has grown that the provision of shelter and care for victims of trafficking among these other populations cannot be tailored to their specific service needs.^1,2^ Therefore, a new programme, called the ‘Categorical Care for Victims of Human Trafficking’ (Dutch acronym: COSM) was established in 2010.^1–5^ ‘Categorical care’ refers to care that is provided exclusively for one population, in this case for victims of human trafficking.

The COSM programme started in June 2010 as a two-year pilot, was extended for two and a half years in 2012, and again in 2014. In 2010 and 2012, three ministries, the Ministry of Health, Welfare, and Sports, the Ministry of Security and Justice, and the Ministry of the Interior and Kingdom Relations,^6^ jointly extended funds for shelter and care services under the COSM programme to three different shelters by means of a public tender. The three shelters that won the tender in 2010 were the same three shelters that won the tender in 2012. In 2014 the Ministry decided to continue care provision through the three shelters that had won the tender in 2010 and 2012.

At the time of the study, the three shelters in the Netherlands that were part of the COSM pilot had a total of 50 beds. Two shelters were for women only and had 20 beds each; one shelter was for men only and had 10 beds. All shelters provide shelter exclusively to adults (and their children). Different shelter arrangements are available in the Netherlands for victims of trafficking who are minors.^7^

In 2012, 113 victims of human trafficking were placed in one of the three COSM shelters. Although a substantial percentage of trafficking victims identified in the Netherlands are of Dutch nationality, the majority of victims who entered the COSM shelters at the time of this research were of foreign nationality (>95%) (later this became 100%, because the entry requirements for the COSM shelters were restricted and only foreign victims were allowed to receive shelter under the COSM programme).^6^ Most victims in the COSM shelters at the time of the research were trafficked for the purpose of sexual exploitation (both women (>95%) and men (75%)) [personal communication T. van Driel from CoMensha d.d. 24 May 2011].

The COSM shelters are a form of crisis shelter. They are intended to provide shelter and care for a maximum of three months. After this, the intention is that victims move towards long-term shelters, supported housing facilities or a house of their own in the Netherlands, or travel back to their home country. The period of three months coincides with the reflection period that victims are entitled to, during which they can decide to cooperate with law enforcement or not (see earlier section “Legislative framework for anti-trafficking efforts in the Netherlands”). There are individual differences between the three shelters, but they all offer secure and safe crisis shelter; facilitate the provision different types of social and health care (either in the shelter or by service providers outside the shelter); and provide information on legal matters and assist service users in acquiring a legal counsellor if required.

## References

1 Londen M van, Hagen L, Brenninkmeijer N. Evaluatie van de pilot ‘Categorale Opvang voor Slachtoffers van Mensenhandel’ (Cahier 2012-14). The Hague, Wetenschappelijk Onderzoek- en Documentatiecentrum (WODC), Ministerie van Veiligheid en Justitie, 2012.

2 Kulu-Glasgow I, Galloway AM, Beenakkers EMT, Smit M, Zwenk F. Categorical Accommodation and Assistance for Victims of Trafficking in Human Beings: A study of four European countries (Cahier 2012-8). The Hague, Wetenschappelijk Onderzoek- en Documentatiecentrum (WODC), Ministerie van Veiligheid en Justitie, 2012.

3 Human Trafficking: Seventh Report from the National Reporter. The Hague, Nationaal Rapporteur Mensenhandel en Seksueel Geweld tegen Kinderen, 2009.

4 Plan van aanpak pilot Categorale Opvang Slachtoffers Mensenhandel (COSM). , 2010.

5 CoMensha. Pilot Categorale Opvang Slachtoffers Mensenhandel van start. 2010.http://www.mensenhandel.nl/cms/index.php?option=com_content&task=view&id=326&Itemid=69.

6 Inkoop: beschrijvend document openbare aanbesteding. Opvang slachtoffers mensenhandel. (kenmerk: 201200117.004.007). Den Haag, Ministerie van Veiligheid en Justitie; Ministerie van Binnenlandse Zaken en Koninkrijksrelaties; Ministerie van Volksgezondheid, Welzijn en Sport, 2012.

7 Kromhout MHC, Liefaard T, Galloway AM, Beenakkers EMT, Kamstra B, Aidala R. Tussen beheersing en begeleiding: Een evaluatie van de pilot ‘beschermde opvang risico-AMV’s’ (Cahier 2010-6). The Hague, Wetenschappelijk Onderzoek- en Documentatiecentrum (WODC), Ministerie van Veiligheid en Justitie, 2010.
