# Supplementary material for: The recovery experience of people who were sex trafficked: the thwarted journey towards goal pursuit
Source: BMC Int Health Hum Rights. 2019 Jan 22;19:3. doi: 10.1186/s12914-019-0185-7 (PMC6341539; doi:10.1186/s12914-019-0185-7)
Supplement: Supplementary file 3 — Context: Topic guides for interviews with service users. This additional file contains the topic guides used for interviewing participants in this research. (DOCX 21 kb) [file 12914_2019_185_MOESM3_ESM.docx]

## Topic guides for interviews with service users

**Non-threatening conversation / getting to know each other**

**Acquiring informed consent and explanation of the research**

- Explaining the purpose of the interview (especially during 1st interview; during 2nd interview check whether purpose is still clear)
- Explain who I am
- Explain interview schedule and times (2 x maximum 1,5 hours but flexible, can be shorter)
- Explain why there is a form to account for fear of forms. Let participant read consent form if their preferred language is Dutch or English, or read out the consent form if the preferred language is any other language and an interpreter has been arranged. Provide additional explanation where necessary. Ask if there are any questions. Participant can sign, mark, or provide verbal consent if he/she agrees to participate.

**Socio-demographics**

- What is your age?
- Where are you from, from which country?
- How long have you been in the Netherlands?
- How long have you resided in this shelter?
- Do you have any children?

**Feelings about previous interview (only during second interview)**

Possible probing questions:

- How do you feel about the previous interview?
- Did you discuss it with others?
- Have you thought about what we discussed during the previous interview?

**Daily lives / personal story**

Possible probing questions:

- Can you tell me a little bit about yourself?
- What does a typical day or week look like for you?
- What did you do yesterday?
- Can you tell me a little bit about what it’s like being here in the shelter^[[1]](#footnote-1)^?
- How have things changed for you here since you arrived?
- What do you hope for, for the future? Are there also things that you worry about? How do you see your future?

**Interaction with other service users**

Possible probing questions:

- Have you made friends here?
- How do service users interact with each other?
- In the shelter where you’re staying there are a lot of service users from different countries. What is it like to live in such a varied group?

**Perceived needs for social and health services**

Possible probing questions:

- Could you explain to me why you are here in the shelter?
- What kind of help do you need from the shelter?
- Why do you need that help?

**Descriptions of service provision**

Possible probing questions:

- How does, or did, the shelter help you with these things?
- What else does the shelter help you with?

**Experiences with services provision**

Possible probing questions:

- Can you give me one or two examples of something that you found really good about the help that the shelter gives you? Why is that good for you?
- And one or two examples of something you found not-so-good? Why is that not-so-good for you? What do you think could be improved?
- If you were the boss of the shelter, what would you change?

**Final questions**

- We discussed a lot of things today. Is there anything that was most important to you, something that I should really pay attention to as part of my research?
- What did you think of the interview(s)? How do you feel about being asked these questions?

**Rounding up**

- Thank participant for his/her participation and for allowing future service users to benefit from this research!
- After the *first* interview, remind service user of the second interview. Ask if the participant would still be happy to take part in the second interview, and if so, if we can arrange a date and time for the next session.
- Also stress that the participant can contact me at any time to talk about what we have discussed today or when there are any questions about the research.
- Thank the interpreter for her/his participation.

1. The shelter is called “xxx” in yyy, “www” in zzz, and “aaa” in bbb. Adjust to formulations used by service users as much as possible. *Names and places of shelters omitted for publication for confidentiality reasons.*  [↑](#footnote-ref-1)
