# Supplementary material for: The recovery experience of people who were sex trafficked: the thwarted journey towards goal pursuit
Source: BMC Int Health Hum Rights. 2019 Jan 22;19:3. doi: 10.1186/s12914-019-0185-7 (PMC6341539; doi:10.1186/s12914-019-0185-7)
Supplement: Supplementary file 4 — Context: Extended description of data analysis methods. This additional file describes in more detail than in the main text the methods used for data analysis in this study. (DOCX 25 kb) [file 12914_2019_185_MOESM4_ESM.docx]

### Extended description of data analysis methods

Data analysis took a broadly grounded theory approach, following the stage of familiarization, open coding, axial coding and selective coding.^1,2^ In addition, some elements of narrative analysis ^3,4^ were incorporated, by writing narratives about service users.^5^ Writing these narratives was an additional way of becoming familiar with the data. These narratives are planned to be published elsewhere.^5^

Data analysis in grounded theory consists of several stages: ^1,2^

1. Familiarization
2. Open coding
3. Axial coding
4. Selective coding

#### Familiarization

Familiarization with the data was conducted through:

- reviewing, synthesizing and digitizing memos made during the interviews, and
- transcribing the interviews

During this stage, additional analytical thoughts, ideas, and other considerations were denoted as memos.

#### Coding, theorizing and writing

Open coding was used in the beginning with the aim of fracturing the data.^1^ Six interviews were open coded. ‘In vivo’ codes were used whenever possible. Open coding was conducted on printed transcripts. At this stage, preliminary links between the codes were also developed (for example, for various codes it was considered what had caused them, what consequences they had, or what smaller codes they consisted of). Toward the end of the open coding stage open codes were consolidated into more definitive codes and categories of codes. This task was performed on a computer using NVIVO versions 9 and 10.

This stage was followed by axial coding in which “one open coding category to focus on (called the ‘core’ phenomenon)” was identified, followed by going back to the data and the creation of categories around the core phenomenon.^2^ In this stage the analysis focused particularly on relationships and links between the various codes, which had already been started preliminarily during the open coding process.

The next stage, selective coding, was to develop further the links between the various codes and the core phenomenon. The goal of this is to “interrelate the categories in the model or assemble a story that describes the interrelationship of categories in the model.” ^2^ This stage consisted of asking a set of questions about the core phenomenon. For example: What were the goals that service users pursued? What was it that drove them to pursue these goals? What was it that held them back? What were the consequences of being thwarted in their goal pursuit? The relevance of these questions will become clear below where the core phenomenon is described.

All stages of coding were primarily performed by the first author; codes, themes and theories that emerged were discussed with the other authors.

#### Writing narratives

As part of the analysis of service users’ interviews, elements of narrative analysis ^3,4^ were incorporated, by writing narratives about service users.^5^ These were written because in the interviews service users often told a story of their lives, speaking of where they had been, their daily lives in the shelters and where they envisioned themselves to be in the future. Writing these narratives was an additional way of becoming familiar with the data.

Each interview was first coded and a summary written about each service user, including a brief description of their baseline characteristics, background, the interviewer’s impressions of the participant during the interview, and key phrases, sections and themes from the interview. Following this, narratives were written about individual service users that reflected the salient themes that emerged from the interviews. These narratives are planned to be published elsewhere.^5^

**References**

1 Green J, Thorogood N. Qualitative Methods for Health Research. London: Sage Publications Ltd, 2005.

2 Creswell JW. Five Qualitative Approaches to Inquiry. In: Qualitative Inquiry and Research Design: Choosing Among Five Approaches. SAGE Publications, Inc, 2007: 53–84.

3 Overcash JA. Narrative research: a review of methodology and relevance to clinical practice. *Crit Rev Oncol Hematol* 2003; **48**: 179–84.

4 Riessman CK. Narrative Analysis. In: Kelly N, Horrocks C, Milnes K, Roberts B, Robinson D, eds. Narrative, Memory and Everyday Life. Huddersfield: University of Huddersfield, 2005.

5 Viergever RF, Thorogood N, Driel T van, Wolf J, Durand MA. Providing shelter and care for victims of human trafficking: service users’ stories (in press). Amersfoort: CoMensha (Coördinatie Centrum Mensenhandel / Coordinating Centre Human Trafficking in the Netherlands), 2018.
